# Gravity stress tibiotalar laxity evaluation with a biomedical gyroscopes device – cadaver study with progressive sectioning of lateral ankle ligaments

**DOI:** 10.1186/s40634-020-00269-z

**Published:** 2020-07-21

**Authors:** F. Guerra-Pinto, J. Cunha, L. Sousa, T. M. Gomes, R. Andrade, X. M. Oliva, J. G. Consciência, P. R. Fernandes

**Affiliations:** 1 Department of Orthopaedics, Hospital Ortopédico de Sant’Ana, R. Benguela 501, 2775-229 Parede, Portugal; 2 Department of Orthopaedics, Hospital da Cruz Vermelha Portuguesa, Lisbon, Portugal; 3 NOVA Medical School, Lisbon NOVA University, Lisbon, Portugal; 4 Department of Anatomy and Human Embryology, Faculty of Medicine, University of Barcelona, Barcelona, Spain; 5 IDMEC, Instituto Superior Técnico, Lisbon University, Lisbon, Portugal; 6 Clínica do Dragão, Espregueira-Mendes Sports Centre – FIFA Medical Centre of Excellence, Porto, Portugal. Dom Henrique Research Centre, Porto, Portugal; 7 Faculty of Sports, University of Porto, Porto, Portugal; 8 Department of Orthopedics, Clinica Del Remei, Barcelona, Spain; 9 Department of Anatomy and Human Embryology, Faculty of Medicine, University of Barcelona, Barcelona, Spain; 10 Head of Orthopaedics Departement at CHLO - S. F. Xavier Hospital, Lisbon, Portugal

**Keywords:** Ankle sprain, Lateral ligaments, Anterior talofibular ligament, Diagnosis device, Instability test, Talocrural joint axis

## Abstract

**Purpose:**

Despite the evidence on the role of gravity stress test to access the instability of other ankle injuries, there is limited literature regarding gravity stress on the lateral ankle ligament’s insufficiency. The objective of our study was to objectively measure the tibiotalar angular movement under gravity stress after progressive sectioning of the lateral ankle ligaments.

**Methods:**

We performed sequential sectioning of the anterior talofibular (ATFL), calcaneofibular (CFL), and posterior talofibular ligaments (PTFL) in twelve ankle specimens. Under gravity stress, we measured the angular movement of the talus in relation to the tibia. The measuring device is based on a three-axis gyroscope and accelerometer.

**Results:**

Comparing to the intact condition, the plantar flexion increased on average 1.78° (95% confidence interval [CI] 1.15;2.42), 5.13° (95%CI 3.10;7.16) and 8.63° (95%CI 6.05;11.22), the rotation increased by 1.00° (95 CI -0.51;2.51), 3.68° (95%CI 1.97;5.40) and 15.62° (95%CI 10.09;21.14), and the varus increased 2.89° (95% CI 1.39, 4.39), 8.12° (95% CI 5.16, 11.07) and 11.68° (95% CI 7.91, 15.46), after sectioning the ATFL, CFL, and PTFL, respectively. The overall changes were statistically significant.

**Conclusions:**

There was a significant tibiotalar laxity after sectioning of lateral ankle ligaments when the foot position is influenced only by gravity. The tibiotalar angular displacement was significant when the CFL and PTFL were cut which suggests that the gravity test could be used to assess combined lateral ankle ligament injury. This evidence might be a step forward in the development of lateral ankle ligaments gravity stress tests.

**Level of evidence:**

5 (cadaver study)

## Background

Ankle sprains are the most common ankle injuries [6]. The ankle is one of the most traumatised anatomical regions during sports activities and accounts for 10–30% of all sports injuries [10]. Ankle sprains can damage several anatomic structures, including the ligaments [19]. Approximately 85% of sprained ankles involve the lateral ligament complex [5]. Lateral ligamentous injury is more prone to injury due to the oblique axis of the subtalar joint that favours supination trauma. In about 65% there is an isolated injury of the anterior talofibular ligament (ATFL) and in 20% both the ATFL and the calcaneofibular ligament (CFL) are involved [5, 6, 10, 19].

The diagnosis of ligament rupture is initially obtained by the clinical history and manual examination that includes inspection, palpation, and instability tests. The manual stress testing for lateral ankle ligaments - the anterior drawer and varus talar tilt tests – are user-dependent and display limited sensitivity. Any method that allows a quantifiable objective measure of the injury severity, discarding the subjectivity of the examiner, can be a significant improvement for the clinical practice. Several devices have been developed to provide more accurate and objective evaluation of ankle ligaments. Among them are the Telos, quasi static anterior ankle tester (QAAT), dynamic anterior ankle tester (DAAT), Ligmaster, Hollis instrumented ankle arthrometer and ankle flexibility tester [4, 7, 9, 11, 12, 14]. The inter-individual variation with the clinical stress testing is not solved by these devices, which suggests that there is room for a different approach.

**Fig. 1:**
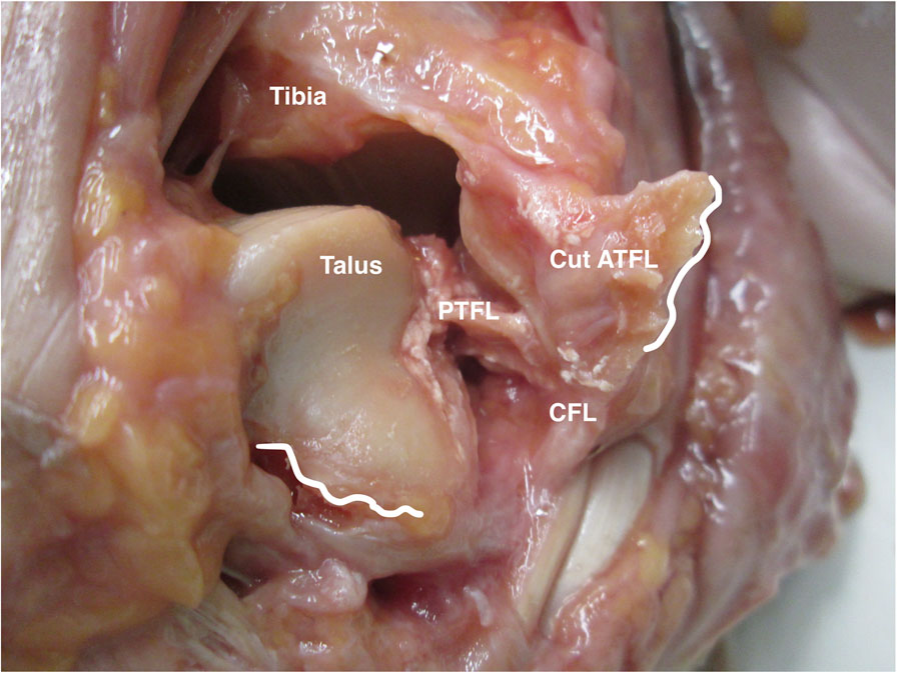
Image of a cadaveric ankle after cutting the ATFL ligaments. The CFL and PTFL are still intact. The white lines show the original position of the ATFL insertion. The gravity stress is enough to show varus and internal rotation of the talus, in relation with the tibia

There has been growing body of evidence in the value of gravity stress tests to evaluate ankle ligaments integrity. The gravity stress test is considered the gold standard in the diagnosis of deltoid ligament injury because of its high specificity and reproducibility [1, 16]. There is also some evidence regarding the use of gravity to diagnose instability in ankle fractures in a seated position [2]. The literature regarding gravity stress testing in lateral ankle ligaments rupture is still scarce. This will require information on the tibiotalar laxity in the three orthogonal planes and should be applied in an experimental setting that replicates the clinical examination. To address this problem, we developed a measuring device with three degrees of freedom for experimental evaluation of sectioning of lateral ankle ligaments (Fig. 1**)**. These degrees of freedom correspond to the rotations in the three anatomical planes (coronal, sagittal and axial) rather than the linear displacement of the talus in relation to the tibia.

The objective of our study was to objectively measure the tibiotalar angular movement under gravity stress according to the damage level (by progressive sectioning) of the lateral ankle ligaments. Our hypothesis is that the gravity stress is enough to produce significant movement of the talus relatively to the tibia after sectioning of the lateral ankle ligaments in an experimental model, and that the corresponding angular variation may be a good objective measure of movement.

## Methods

### Device development: hardware

**Fig. 2:**
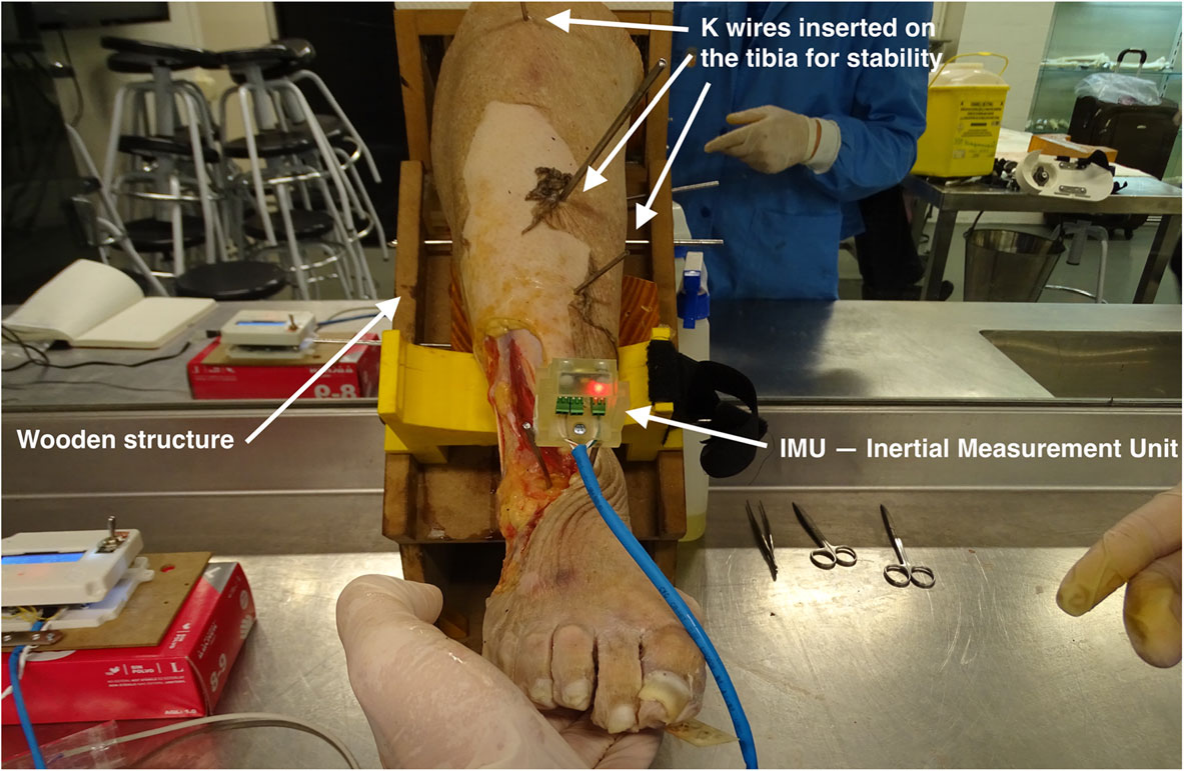
Description of the device components. Image of the experimental setting on a specimen

A support was designed and built in medium density fiberboard (MDF) wood to hold the lower limb. For high reliability on readings three Kirschner wires (3 mm diameter) were inserted in the tibia and fixed to the MDF structure in order to immobilize it, avoiding any rotation of the lower limb, and allowing only the movement of the ankle joint (Fig. 2**)**. This support holds the leg in a 45 degrees angle with the horizontal plane.

The measurement system comprised a Mpu-6050 GY-521, which is a 6 degrees of freedom inertial measurement unit (IMU) with three-axis accelerometer and a three-axis gyroscope to obtain the movement angles. The IMU must be fixed to the talus. Two Kirschner wires (3 mm diameter) were inserted in the talus neck in an anterior-posterior direction, intersecting its longitudinal axis and the IMU’s were fixed to the wire making both the talus and the IMU rigidly connected. The IMU sensor is controlled by an Arduino Mega 2560 board, which was used as the microcontroller. The calibration of the system assured that the gyroscopes will measure the rotation angles (yaw, pitch and roll) between the starting position and final movement position after each step of the testing protocol. This way, it was possible to do a precise evaluation of the tibiotalar movements without interference of hind-foot, mid-foot or fore-foot movements. Only the rotations of the talus were being measured with respect to its three own cardinal planes (axial, coronal, and sagittal).

### Device development: software

A sensor fusion algorithm was used for the acquisition and interpretation of the raw data received from the IMU. The programming code is based on open source libraries available on internet, performing the initialization, calibration (including offset values) and filtering of the sensor values. This process ensures more accurate values of the relative angles. A new dedicated software was developed, which enabled us to obtain the angular values relative to the difference between the initial orientation of the ankle’s body fixed axes and the final orientation originated by extrinsic rotations. The software allows real time analysis of the angular displacement of the talus in three planes, simultaneously, using Tait–Bryan angles.

**Fig. 3:**
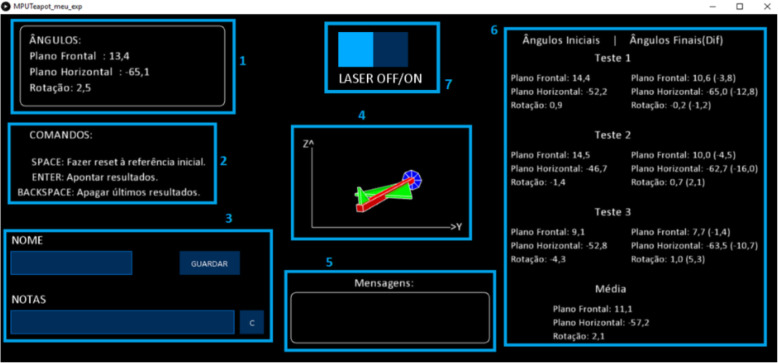
Software user interface. This layout is a computer screenshot during the experimental setting. Gyroscopes and accelerometers are used in commercial aviation and work like a 3-dimensional compass, showing the position of the airplane relative to the ground. For this reason the Figure in the centre of the screen, which represents the foot position, is displayed as an airplane. On the upper left box, “Ângulos” stands for angles; “Plano Frontal” refers to the coronal plane measurement; “Plano Horizontal” refers to the sagittal plane measurement and “Rotação” refers to the axial plane

The software interface has some features that can be very helpful with the device such as real-time angle measurement, entries for the name and observations to save the data in an excel document and 3D visualization of the movement. This user interface intends to help the user with a graphical context and is divided in seven regions (Fig. 3). It was developed in “Processing” (an open source programming language based on graphical representation, https://www.processing.org/) to communicate with Arduino. The user can observe the angle values change in real time (region 1) along with its visual movements (region 4), perform a set of tests (region 6) and save the results (region 3) in a “.tsv” file (tab separated values) to be read into a spreadsheet Excel file for data analysis. Each time the user presses the “Enter” key the values shown on region 1 are copied to region 6. In region 7 the user can switch the laser on/off.

### Cadaveric tests

Twelve specimens were obtained under to the body donation program from the Institution where the cadaveric tests were performed. It complies with all bioethical requirement according the rules of this donation program approved by our University. It comprised 7 female and 5 male patients with ages comprehended between 57 to 81 years old (average 72).

All specimens were frozen no more than 1 year and were defrost to be used in this study according to the guidelines of the pre-existent local program. The twelve cadaveric ankles were free of any abnormalities. The tibia and fibula were systematically sectioned below the knee joint. The skin and fat pat over the distal third of the leg and ankle were removed. The proximal and distal extensor tendon retinaculum were also removed allowing the access to the anterior joint line, whereas the peroneal tendon’s retinaculum was opened longitudinally. The ankle joint was opened in the mid-line by detaching the anterior capsule from the distal tibia, while preserving the medial and lateral capsule-ligamentous complex. No tissue was removed around the anterior talofibular and the calcaneofibular ligaments, because the anterolateral capsule complex might have some mechanical resistance.

**Fig. 4:**
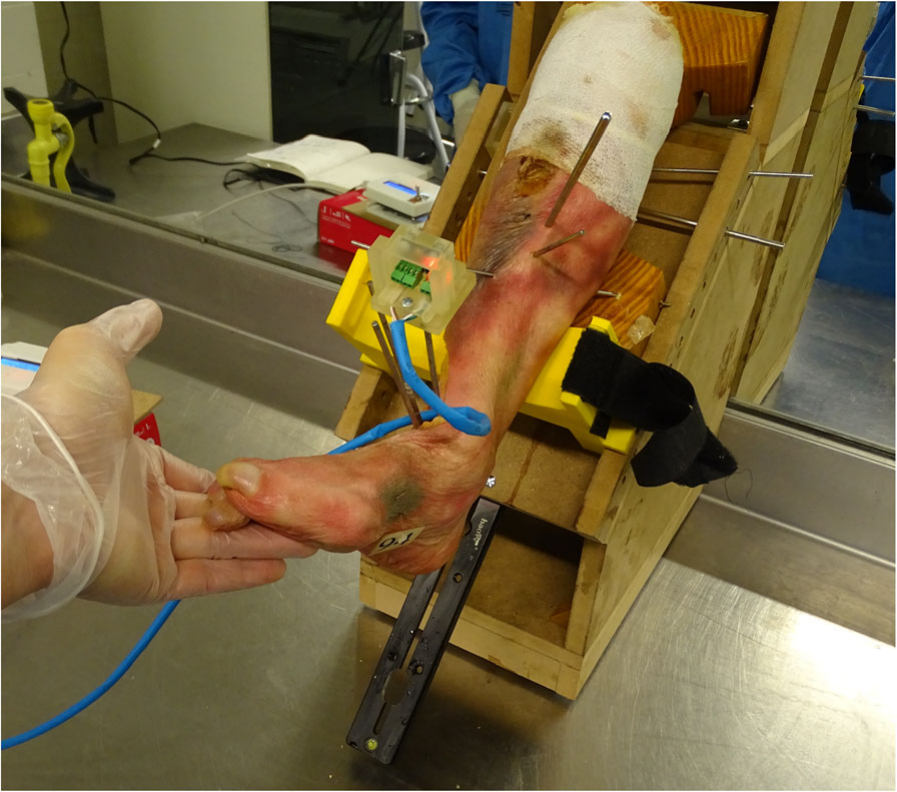
Ankle in a neutral position for system calibration

**Fig. 5:**
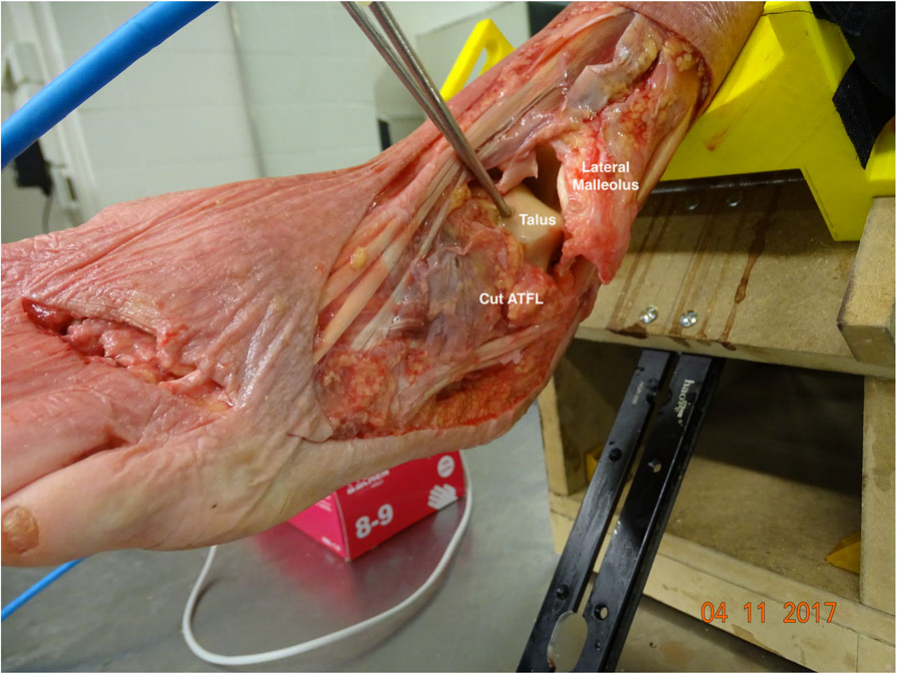
Cadaver model after ATFL, CFL and PTFL section. The cut ATFL and antero-lateral capsule remnants are visible. There is evident subluxation of the talus relative to the ankle mortise

**Fig. 6:**
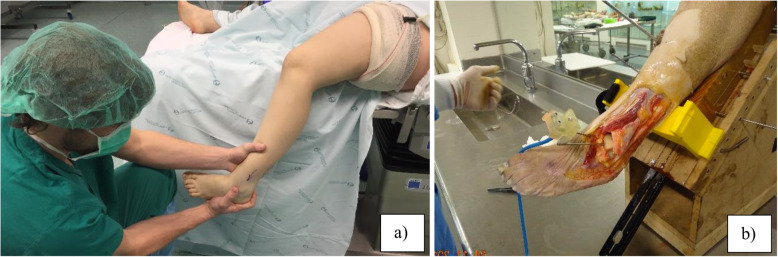
**a** Clinical picture of a hanging leg during ankle examination. During this maneuver the leg is oriented 45 degrees in relation to the floor; **b** Cadaver model after lateral ankle ligaments section (ATFL+CFL + PTFL). The leg orientation is similar to the clinical objective examination

The tibiotalar displacement was evaluated through the prototyping device described above. For each specimen our research started by calibrating the system in neutral ankle dorsiflexion by holding the foot at 90° to the tibia, which means a square ankle, and the tibia oriented 45° in relation to the floor/table where it stood. That was our zero reference and initial position (Fig. 4). The 90° angle is assured by using a set square, and the 45° angle is imposed by the wood support of the device. This tibia orientation is similar to the leg position during regular clinical examinations, when the patient is lying on a bench and the hanging foot and ankle is held by the medical examiner (Figs. 5 and 6).

We would then let the gravitational forces act on the hanging foot and record its final orientation (Fig. 5). It should be noticed that the starting position is the same as the one used in the clinical objective examination, as shown if Fig. 6b. Four different measurements were taken. On each specimen, the first measurement was done with all ligaments intact and the followings after progressive sectioning each of the lateral ankle ligaments (ATFL, CFL and PTFL). The second measurement was done after a complete section of the ATFL. This was done through a complete section of the capsule and ligament from the anterior facet of the lateral malleolus. The third measurement was done after a complete section of the CFL from the inferior aspect of the lateral malleolus. The fourth measurement was done after complete section of the PTFL. This section was done from anterior to posterior, through the ATFL section window into the PTFL, as well as behind the lateral malleolus while reflecting the peroneal tendons posteriorly. At each measurement point the value of the angular rotations was registered in the three planes of movement: sagittal (flexion), axial (rotation) and coronal (varus).

### Statistical analysis

All statistical analyses were conducted using the IBM SPSS Statistics V.24.0. A significance level of 0.05 was considered for all statistic tests. Continuous variables were described with described using mean and standard deviation (SD). The variables were tested for outliers and normality (Kolmogorov-Smirnov test). Mean differences (angular displacement) with the respective 95% confidence intervals (CI) were calculated as the difference of intact and ligament sectioning condition. The Friedman test was used to test the overall changes and the Bonferroni correction was used to compute the adjusted significance between pairwise comparisons of progressive sectioning. The Kendal W was computed to evaluate the consistency of changes. The intraclass correlation was used to test the variability of the different measures based on a single measure, absolute-agreement, 2-way mixed-effects model. Values less than 0.5 are indicative of high variability, values between 0.5 and 0.75 indicate moderate variability, values between 0.75 and 0.9 indicate low variability, and values greater than 0.90 indicate very low variability [13]. The standardized response mean (SRM) and effect size (ES) were used to measure the responsiveness of progressive sectioning. The SRM was defined as the mean change between the two conditions divided by the standard deviation (SD) of this change. The ES was defined as the mean change between intact and sectioning conditions divided by the SD of the intact condition. The ES and SRM were considered large if greater than 0.80, moderate if between 0.51 and 0.80 and small if lower than 0.50 [3].

## Results

**Fig. 7:**
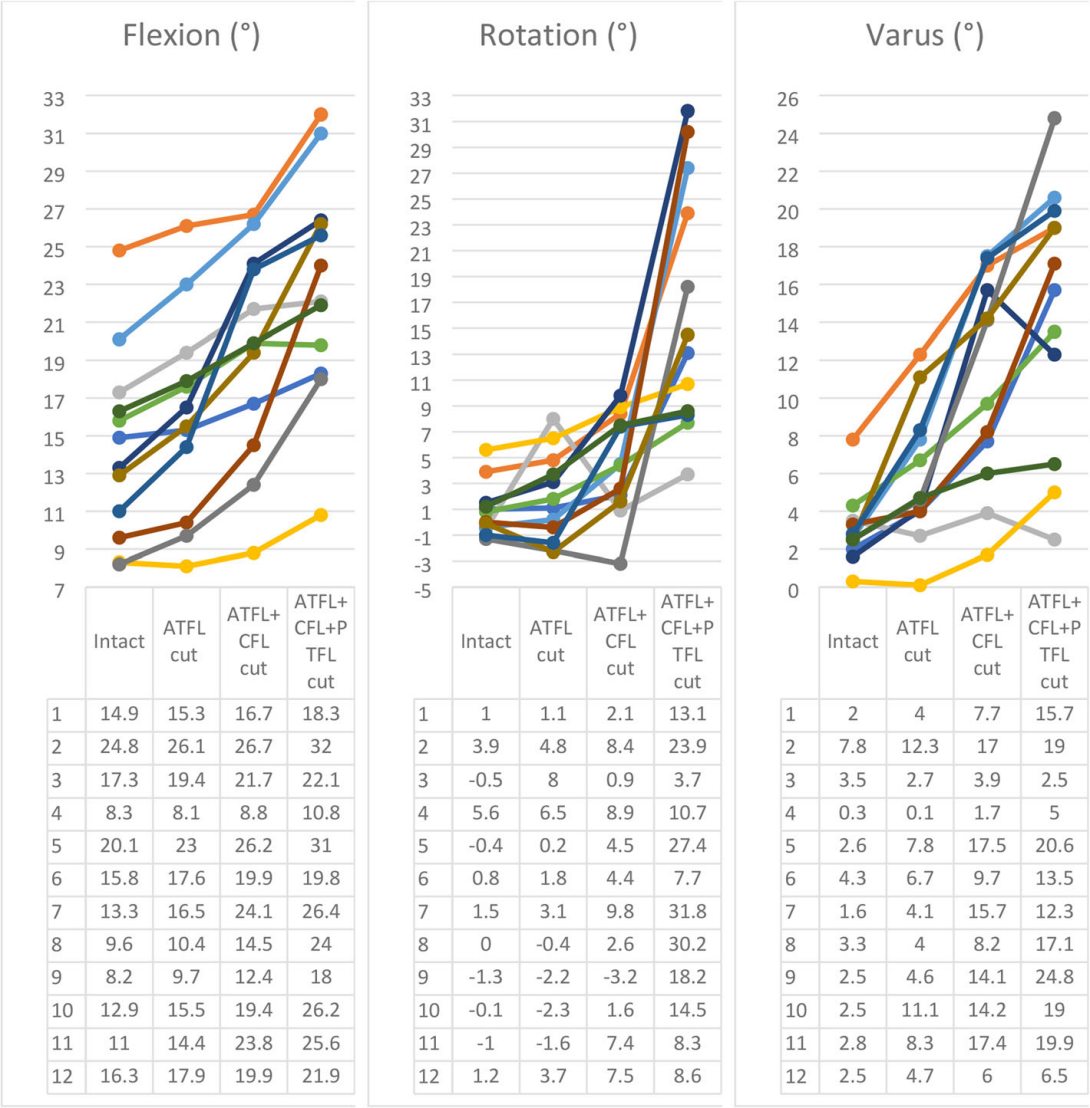
Amount of flexion, rotation and varus exhibited by the foot after sequential sectioning

The results obtained can be seen in the Fig. 7. Each graph shows the degrees of the talus angular displacement in relation to the tibia, on the three cardinal planes, during the four sequential experimental steps: (1st) intact ligaments, (2nd) ATFL section, (3rd) ATFL and CFL section and (4th) ATFL, CFL and PTFL section.

Figure 7a shows the amount of plantar flexion. A positive angle means plantarflexion and a negative means dorsiflexion. After sectioning the ATFL, CFL, and PTFL, talus plantar flexion increased on average 1.78° (95% CI 1.15, 2.42), 5.13° (95% CI 3.10, 7.16) and 8.63° (95% CI 6.05, 11.22), respectively. The Fig. 7b shows the amount of rotation exhibited by the talus in the same terms of flexion. A positive angle means external talus rotation and a negative one means internal rotation in relation to the fixed tibia. After sectioning the ATFL, CFL, and PTFL, rotation increased 1.00° (95% CI -0.51, 2.51), 3.68° (95% CI 1.97, 5.40) and 15.62° (95% CI 10.09, 21.14), respectively. Figure 7c shows the amount of varus exhibited by the talus in similarity to both flexion and rotation. A positive angle means varus and a negative angle means valgus. After sectioning the ATFL, CFL, and PTFL the varus increased on average 2.89° (95% CI 1.39, 4.39), 8.12° (95% CI 5.16, 11.07) and 11.68° (95% CI 7.91, 15.46), respectively. Overall changes significantly different from intact position, showing good consistency (Kendal’s W 0.717 to 0.939). Changes from intact condition were significant for flexion when sectioning the ATFL+CFL and the ATFL+CFL + PTFL, for rotation when sectioning the ATFL+CFL + PTFL, and for varus when sectioning ATFL+CFL and the ATFL+CFL + PTFL. None of the progressive changes of progressive sectioning were significant (Table 1**).** Flexion and varus measurements showed moderate variability and moderate to large responsiveness. The talus rotation measurements had high variability, high variability of responsiveness: low and moderate responsiveness when the ATLF and the ATFL+CFL were cut, respectively, but large responsiveness when the ATFL+CFL + PTFL were cut (Table 2).

**Table 1:** Changes of progressive ligament sectioning

	Flexion			Rotation			Varus		
**Change from intact condition**	**(ATFL cut - intact)**	**(ATFL + CFL cut - intact)**	**(ATFL + CFL+ PTFL cut - intact)**	**(ATFL cut - intact)**	**(ATFL + CFL cut - intact)**	**(ATFL + CFL + PTFL cut - intact)**	**(ATFL cut - intact)**	**(ATFL + CFL cut - intact)**	**(ATFL + CFL + PTFL cut - intact)**
**MD (95% CI)**	1.78 (1.15, 2.42)	5.13 (3.10, 7.16)	8.63 (6.05, 11.22)	1.00 (−0.51, 2.51)	3.68 (1.97, 5.40)	15.62 (10.09, 21.14)	2.89 (1.39, 4.39)	8.12 (5.16, 11.07)	11.68 (7.91, 15.46)
**Bonferroni correction**	0.683	**0.001**	**< 0.001**	1.000	0.068	**< 0.001**	1.000	**0.002**	**< 0.001**
**Progressive changes**	**(ATFL cut - intact)**	**(ATFL + CFL cut - ATFL cut)**	**(ATFL + CFL+ PTFL cut - ATFL + CFL cut)**	**(ATFL cut - intact)**	**(ATFL + CFL cut - ATFL cut)**	**(ATFL + CFL + PTFL cut - ATFL + CFL cut)**	**(ATFL cut - intact)**	**(ATFL + CFL cut - ATFL cut)**	**(ATFL + CFL + PTFL cut - ATFL + CFL cut)**
**MD (95% CI)**	1.78 (1.14, 2.41)	3.35 (1.84, 4.86)	3.50 (1.87, 5.13)	1.00 (−0.51, 2.51)	2.68 (0.43, 4.94)	11.93 (6.35, 17.52)	2.89 (1.39, 4.39)	5.23 (3.12, 7.33)	3.57 (1.23, 5.90)
**Bonferroni correction**	0.683	0.162	0.683	1.000	0.683	0.161	1.000	0.106	1.000
**W Kendal**	W = 0.939			W = 0.717			W = 0.742		
**Friedman**	***p*** **< 0.001**			***p*** **< 0.001**			***p*** **< 0.001**		

Bold values indicate statistical significance. *MD* mean difference, *CI* confidence interval, *ATFL* anterior talofibular ligament, *CFL* calcaneofibular ligament, *PTFL* posterior talofibular ligament

**Table 2:** Reliability and responsiveness of progressive ligament sectioning

Motion	Condition	ICC (95% CI)	Effect Size	SRM
**Flexion**	**ATFL cut - intact**	0.813 (0.624, 0.933)	0.361	1.592
	**ATFL + CFL cut - ATFL cut**		0.636	1.255
	**ATFL + CFL + PTFL cut - ATFL + CFL cut**		0.630	1.215
**Rotation**	**ATFL cut - intact**	0.067 (−0.132, 0.427)	0.492	0.375
	**ATFL + CFL cut - ATFL cut**		0.790	0.674
	**ATFL + CFL + PTFL cut - ATFL + CFL cut**		3.035	1.209
**Varus**	**ATFL cut - intact**	0.506 (0.218, 0.787)	1.593	1.089
	**ATFL + CFL cut - ATFL cut**		1.489	1.406
	**ATFL + CFL + PTFL cut - ATFL + CFL cut**		0.638	0.865

## Discussion

Our study showed that the gravity stress causes significant tibiotalar angular displacement when the lateral ankle ligaments are progressively sectioned. The tibiotalar angular displacement was significant mostly when the ATFL+CFL and ATFL+CFL + PTFL were cut comparing to the intact condition, but progressive changes were not significant. This finding suggests that the gravity test could be used to assess combined lateral ankle ligament injury, but not to identify isolated ligament injuries nor to differentiate which ligaments are injured. This evidence fills a gap in literature regarding the experimental basis for the lateral ankle ligaments gravity stress testing.

To the date, most ankle arthrometers tried to quantify the anterior displacement of the talus during an anterior drawer test. Some of these available devices are also able to measure the angular displacement around a single axis [4, 9, 14]. Inconsistencies in the anterior drawer test and on the varus talar tilt stress tests might be due to the fact that insufficiency of the lateral ankle ligaments is mostly caused by a supination trauma and has an instability vector which is hard to simplify to just one of the orthogonal planes [4, 7, 8, 11, 12, 14, 15, 17, 18].

Most of the existing devices performed the measurements with the foot resting in a holding platform and do not allow simultaneous measurement of the angular displacement in several axes. To overcome this difficulty in the ankle instability evaluation, namely the inconsistency between the traditional diagnosis manoeuvres and clinical results [8, 20], a new experimental model is proposed in this work. The objective is to obtain a methodology to objectively quantify the movement of the ankle depending on the damage level without any interference, except gravity.

We found inter-individual variation in all movements. We could observe the increasing laxity, but there were differences between the physiological laxities of each specimen on the different test phases. The variability was more pronounced when measuring the rotation, showing poor reliability and responsiveness. This finding could be due to the differences in internal/external talus rotation, that change the movement between positive (external rotation) and negative (internal rotation). During plantarflexion movement, the natural path the foot takes without externally applied forces seemed to be unique being this more obvious when all the ligaments were intact, i.e. letting the foot hang from the neutral positions brought the talus to a similar position throughout each of the twelve cadaveric ankles. This finding is supported by Leardini et al. [15], who stated that when the ligaments are intact, the articular surfaces constrain a preferred path of joint motion. From the neutral position, the natural joint movement during plantarflexion showed a slight internal rotation in about half the specimens after ATFL section, but this axial plane movement changed to an external rotation in almost all ankles after a double (ATFL plus CFL) ligament section. This external rotation is, in our opinion, related to the articular incongruency and subluxation only possible after double (ATFL plus CFL) lateral ligament lesion. The ATFL is the main stabilizer against internal talus rotation. Adding CFL section will result in a varus talar tilt, unblocking the talus from its mortise and allowing that the gravity forces put an external rotation spin on the talus. Without the incongruency in the coronal plane the talus cannot externally rotate in the mortise, unless there is a lateral malleolus fracture or a syndesmosis rupture.

The major limitation of the study is its specificity for cadavers, due to the insertion of the Kirshner wires in the bony surfaces. However, it is possible to reproduce the results on individuals with slight modifications to the device (reflective markers instead of Kirshner wires) and validate these findings in the clinical practice. The foot movement was induced by gravity, but the angular displacements after progressive ligament sectioning are similar for most tested ankles. As we did not repeat the measurements for the same cadavers and same condition, the test-retest reliability could not be assessed. We also did not compare our measurements with a gold-standard device because it was not available, and thus could not test the validity of the device. Further studies are needed to ascertain the reliability and validity of our device to assess lateral ankle ligament laxity in human specimens. There are intrinsic limitations of experimental cadaveric tests, most importantly, the role of dynamic stabilizers that is annulled. For the purpose of isolated evaluation of passive stability this is optimal, but with poor translation into real conditions. Our sample size was small (12 cadavers), which is common to most cadaveric studies.

The major advantage of performing this study in cadavers is the possibility of having a sensor rigidly connected to the talus through an invasive preparation thus providing the angular displacement between two bones with precision. This type ok kinematic bone-to-bone movement measurement was the choice of many authors, such as the work of Gehring et al. [7]. Although our rational makes the use of 3D evaluation like Wenning et al. [21], it has an original approach due to the unload foot and measurement of the pathologic laxity under gravity stress. It is expected that results would be slightly different if the sensor was placed on the surface of the foot due to the relative movements of the soft tissues between the bones and the sensor, but it would still be able to make the measurements.

The strengths of our research are the proposed methodology and the developed device which are original and a promising tool to quantify the ankle laxity. Measurements were made using a three-axis gyroscope and accelerometer and registered through a computer interface developed for use of an Arduino microcontroller. This represents a departure from existing methods in two main aspects: our device measures three-dimensional angular variations and the foot is only subject to gravity parting from an initial position that correspond to the position used by clinicians to do the objective examination. In fact, it is possible to have a continuous measurement of the foot rotation, although we only recorded the stable ones before and after sectioning the ligaments. Our device allows very precise measurements (up to centesimal degree) of ankle angular movements and shows high reproducibility and responsiveness.

## Conclusions

There was a significant increase tibiotalar laxity (plantar flexion, talus rotation and varus) after progressive sectioning of lateral ankle ligament in which the examiner does not apply any ankle stress and the foot position is influenced only by gravity. The tibiotalar angular displacement was significant mostly when the CFL and PTFL were cut comparing to the intact condition, but progressive changes were not significant, which suggests that the gravity test could be used to assess combined lateral ankle ligament injury. This evidence might be a step forward in the development of lateral ankle ligaments gravity stress tests.
